# Pathogenicity and virulence of Powassan virus

**DOI:** 10.1080/21505594.2025.2523887

**Published:** 2025-06-22

**Authors:** Manpreet Kaur, Monica Adam, Megan C. Mladinich

**Affiliations:** Department of Biological Sciences, SUNY Old Westbury, Old Westbury, NY, USA

**Keywords:** Powassan virus, Flavivirus, age-dependence, neurovirulence, virulence factors, encephalitis, microgliosis, neuroinflammation, tick-borne

## Abstract

Powassan viruses (POWV) are emergent tick-borne Flaviviruses that cause severe encephalitis and long-term neurologic sequelae in patients. POWVs are rapidly spread in the saliva of *Ixodes* tick species during a 15-minute tick bite. In recent years, there has been an increased incidence of severe POWV disease in the Northeastern United States and seroprevalence studies suggest that POWV is underreported. Concurrently, the geographic range of vector tick species has and continues to expand, suggesting that POWV incidence in the US will only increase. Despite the expanding geographic range of vector ticks and increased incidence of POWV disease in recent years, there are currently no licenced vaccines or therapeutics for treating POWV disease. Here, we review the current literature and understanding of POWV with an emphasis on POWV epidemiology, pathogenesis, virulence factors and host immune responses. We also highlight gaps in the current POWV knowledge and directions for future POWV research efforts.

## Introduction

Powassan viruses are emergent tick-borne Flaviviruses (FV) associated with severe encephalitic disease and long-term neurologic sequelae [1–3]. FVs are enveloped, positive-sense single-strand RNA viruses largely transmitted by arthropod vectors such as ticks or mosquitos [4]. Arthropod-borne FV infections (eg. Dengue, Zika, West Nile, and tick-borne encephalitis virus) are an ongoing global public health concern, with an estimated 400 million cases annually and epidemic potential [4]. Reliant on vector species for transmission, arthropod-borne FVs are controlled by vector species’ life cycle, seasonality, and geographic range [5]. In recent years, changes in environmental factors such as climate and host availability have resulted in the expansion of POWV *Ixodes* tick vector species’ geographic distribution [1,6]. Consistent with an increasing vector range and despite being underreported, POWV case incidence continues to rise slowly across North America. POWV-infected patients report a range of symptoms from mild-febrile illness to neurodegenerative disease, with the most severe disease presentations and fatalities in individuals >60 years old [1,7–10]. Currently, there are no licenced prophylactics or therapeutics to treat neuroinvasive POWV in patients. An improved understanding of POWV virulence and pathogenesis from the tick bite site to the brain is required for the development of POWV-specific therapeutic targets. Here, we review current POWV research with a focus on POWV epidemiology, pathogenesis, virulence factors, and host immune responses. Further, we assess gaps in the field for the scientific advancement of POWV research and development of POWV therapies and vaccines.

## Epidemiology

### POWV disease

**Figure 1.**
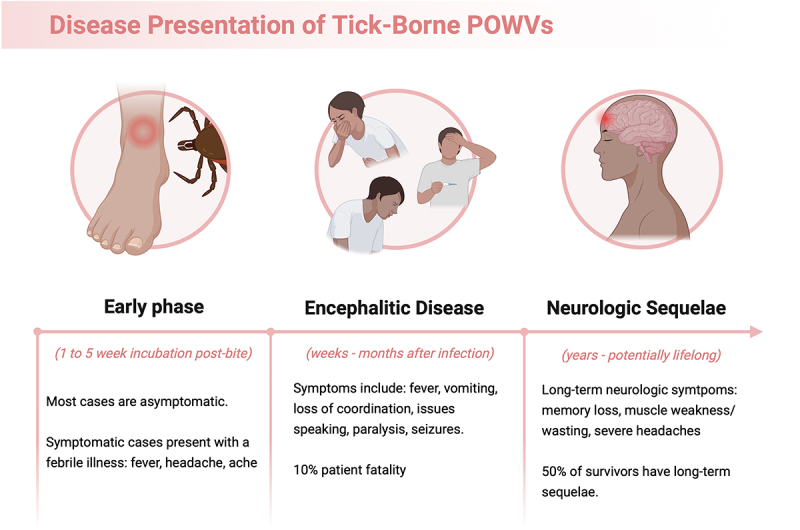
Powassan virus disease presentations in patients. Created in BioRender. (2025).

POWV infections occur following the bite of *Ixodes* tick vector species. Most POWV cases are asymptomatic, and ~ 20% of arthropod-borne FV infections result in an acute febrile illness [4]. However, encephalitic POWV cases can result in neurologic diseases that can last months to years [11] (Figure 1). Severe POWV infections are biphasic, with acute symptoms beginning 1–5 weeks post-tick bite, followed by encephalitis that can persist for several weeks to months [8,11,12]. Symptoms of POWV encephalitis may include muscle weakness, memory loss, and seizures as a result of long-term neurologic harm [13]. POWV-induced encephalitis may also progress into severe CNS damage, including gliosis in the cerebral cortex, which is potentially fatal [1,8,11]. The fatality rate of POWV-infected patients is approximately 10%. Further, 50% of individuals who survive experience long-term neurological sequelae [1,2,11,14]. Individuals of all age groups are at risk of POWV infection. However, data suggests that patients above the age of 60 may be at a greater risk of more severe neurologic deterioration [1,7–10,14]. In endemic states where POWV is more common, between 0.7% and 6.1% of the population show serological evidence of exposure as indicated by the presence of POWV antibody titres. Despite this, fewer clinical cases have been reported, suggesting that POWV infections are largely asymptomatic, making the tracking of this disease particularly challenging [1,7,8,12].

### Powassan viruses

POWV has two distinct genetic lineages, Lineage I (POWV-LB) and Lineage II (POWV-Spooner, Deer-tick virus, POWV-Long Island 9), spread by different species of ticks [14–17]. Lineage I POWVs are primarily associated with *lxodes cookei* ticks, whereas lineage II POWVs are associated with *lxodes scapularis* ticks [18]. Despite being spread by separate vector species, POWV lineages I and II appear to cause similar diseases in mice and humans. Lineage I and II envelope (E) proteins are 96% identical, and nucleic acid sequences are 86% identical. This high degree of similarity renders them serologically indistinguishable [15,16,19]. Their relatedness is valuable as it suggests a comprehensive efficacy potential for POWV vaccines [20].

### POWV distribution and vectors

POWV is an enzootic virus found in parts of North America and Canada but there have been cases reported in parts of Russia and Asia as well. In North America, the enzootic cycle is primarily maintained in small animals such as squirrels, groundhogs, white-footed mice, and skunks [21]. Humans who live in areas with vector species and animal hosts are at higher risk for tick-borne disease. In recent years, monitoring of POWV vector tick species has revealed an expanding geographic range coincident with a significant increase in reported human cases within the last two decades [1,6,14,22,23]. There has been an increasing concern that climate change may encourage an expansion of the geographic range of POWV tick vectors [4,23–25]. For example, as temperatures continue to rise, *lxodes cookei* ticks are expected to travel north towards Canada [26]. Additionally, the momentum of urbanization has contributed to habitat fragmentation, forcing populations of tick hosts to migrate and, therefore, exposing tick-borne diseases to new ecosystems. As hosts migrate away, biodiversity decreases in fragmented environments. Hosts that can tolerate environmental changes like urbanization, such as the white-footed mice, gather in these fragments of land. This creates focal points for POWV, increasing the likelihood of tick circulation in those areas [26].

### POWV case trends

The first documentation and isolation of POWV was in 1958 in Powassan, Ontario. POWV was isolated from the brain of a young boy who passed away from encephalitic disease [17]. Over the 50 years from 1958 to 1998, there were 27 total cases reported in North America [7,14,18]. Since then, there has been an upward trend in the average yearly cases in North America, with 54 cases reported in 2024 in the United States alone [27]. More concerningly, ~90% of reported cases are neuroinvasive, and are in locations that had not previously reported POWV [9,18]. POWV cases continue to rise, likely due to increased vector range, increased human exposure, and improved monitoring of POWV. Regardless, rising POWV cases in North America call for a better understanding of POWV pathogenesis.

## Pathogenesis

### POWV replication at the bite site

**Figure 2.**
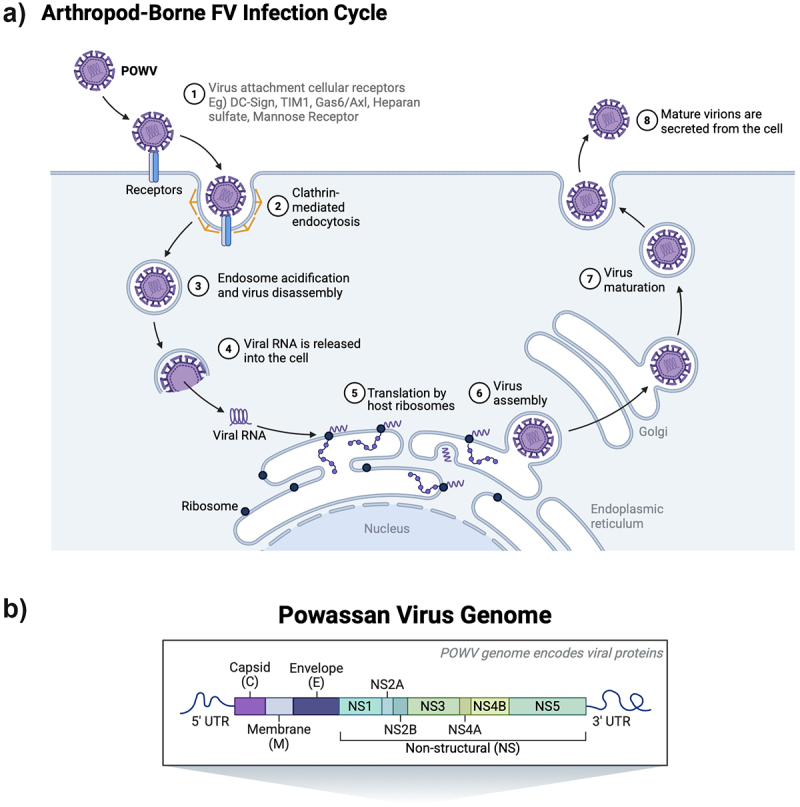
Arthropod-borne flavivirus replication and genome. (a) flavivirus replication created in BioRender. (2025). (b) Powassan virus genome encodes viral proteins. Created in BioRender (2025).

POWV spreads from an *Ixodes* tick to the host through tick saliva in as little as 15 minutes [14,28,29]. POWV transmission is rapid compared to other tick-borne pathogens, such as the 36–48 hours taken to transmit *Borrelia burgdorferi*, the causative agent of Lyme disease [30]. At the bite site, the FVs infect permissive cells, entering cells via receptor-mediated endocytosis [4,31–33]. Cellular targets at the bite site of tick-borne FVs are thought to include keratinocytes and dendritic Langerhans cells [33–36]. Following endocytosis, acidification in the cellular endosome stimulates the fusion of the viral envelope with the endosome membrane and the release of the viral genome into the cytoplasm of the cell (Figure 2a). Host cell ribosomes translate the FV genome in the cytoplasm and the capsid protein-RNA complex buds into the lumen of the ER, acquiring the virion membrane and Envelope surface proteins [4,37,38]. The FV positive-sense single-strand RNA genome is ~ 11kb and contains one open reading frame (ORF) that encodes all the viral proteins (Figure 2b). During and following translation, the FV polyprotein is cleaved by viral and host proteases to form three structural proteins (Capsid, pre-Membrane, and Envelope) and seven nonstructural proteins (NS1, NS2A, NS2B, NS3, NS4, and NS5) [4,39,40] involved in replication and viral assembly. Viral structural proteins and RNA are assembled into immature viral particles in the lumen of the ER and move to the Golgi apparatus through a secretory mechanism [41]. In the Golgi, immature viral particles are processed further by host furin proteases, and the mature FV particles are secreted from the cell via an aberrant secretory mechanism [42].

Low levels of POWV viraemia begin soon after infection at the bite site. Following subcutaneous infection of C57BL/6 mice, POWV-LB RNA was detected in lymph nodes within 24 hours [43,44]. Similarly, Lineage II DTV-infected mice had low levels of POWV present in the serum by 1–3 days post-infection [45,46]. POWVs have been shown to haemagglutinate, which may promote viral dissemination via red blood cells [14]. In addition, macrophages and fibroblasts detected at the bite site 3–24 hp bite were positive for POWV-LB antigen [14,44,47]. It is unclear if these cells are infected or have phagocytosed and eliminated POWV particles, but they may contribute to viraemia and neuroinvasion. Following an undetermined dissemination route, POWVs are detected in the CNS ~5 days post POWV inoculation of C57BL6 mice [43,48].

### POWV neuroinvasion

**Figure 3.**
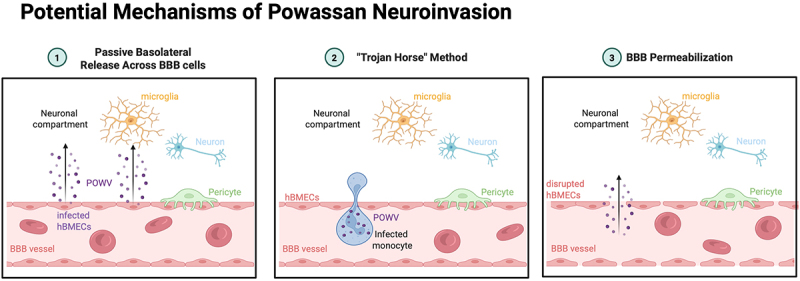
Potential mechanisms of Powassan virus neuroinvasion. Created in BioRender. (2025) https://biorender.com/h83l005.

Neuroinvasion is a critical step in POWV disease progression to severe encephalitis. The mechanisms of POWV neuroinvasion have not been defined. The blood-brain barrier (BBB) complex protects the central nervous system and comprises human microvascular endothelial cells (hBMEC), pericytes, and astrocytes [15,49]. There are several possible means by which neurovirulent FVs can gain access to the neuronal compartment, including in infected immune cells, passively via infection of the BBB or by actively disrupting the BBB endothelial cell tight junctions [50,51] (Figure 3). Immune cells access privileged compartments, and therefore, infected immune cells can act as a Trojan horse carrying the virus directly across the BBB [51]. POWV could potentially infect immune cells such as dendritic cells, monocytes, or macrophages at the site of the tick bite and traverse the BBB in these cells [52–54]. As mentioned, macrophages at the tick bite site were positive for POWV RNA 3–24 hours post-infection, suggesting this as a potential POWV neuroinvasion mechanism [44,47]. FV can also passively cross the BBB through infection of BBB endothelial cells [15,51,55]. A study using POWV-LI9 found that POWV infects and persists in primary hBMECs and pericytes *in vitro*. Transwell assays indicate that hBMECs are not permeabilized by POWV-LI9 infection, and POWV is preferentially released basolaterally. The basolateral release suggests a mechanism for POWV to be released passively across the blood-brain barrier to the CNS [15]. However, this CNS entry mechanism has not been studied *in vivo*. Some neurotropic viruses cross the BBB after causing dysregulation of the tight junctions enhancing BBB permeability [56]. TBEV and WNV have been shown to permeabilize the BBB, which would suggest another potential mechanism of closely related POWV traversing the BBB [57,58]. However, POWV-LI9 does not appear to permeabilize hBMECs *in vitro* [15]. Lastly, an alternate pathway has been proposed for TBEV and WNV CNS infection. TBEV and invasion of olfactory epithelium, is thought to lead to infection of olfactory neurons and result in TBEV neuroinvasion. However, POWV very rarely infects the olfactory bulb [14,45,50,51].

### POWV neuropathogenesis

Neuroinvasive FV like TBEV, WNV, JEV, and POWV cause CNS diseases including encephalitis, meningitis, and paralysis, and can result in death [59]. Similar to POWV, TBEV causes an acute febrile illness followed by lethal encephalitis and 40% long-term neurologic damage [60,61]. In humans, TBEV antigen is detected in neurons and lesions in the brainstem, cerebrum, cerebellum, and thalamus [62–65]. In mice, POWV-induced neuropathology is also detected in these regions, especially the cerebellum, spinal cord, and brainstem [45,48]. Pathologies in these regions of the brain are consistent with POWV disease presentations, including seizures and paralysis [45]. In C57BL/6 mice, POWV-LB (Lineage I) infection of the CNS presents as meningoencephalitis with mononuclear cell infiltration, gliosis, and POWV antigen detected in the spinal cord, thalamus, and cerebellum [14,44]. Similarly, POWV-LI9 (Lineage II) CNS infection of C57BL/6 mice is characterized by spongiform encephalopathy, immune cell infiltration, gliosis, and POWV RNA detected throughout the brain [48]. For both TBEV and POWV, more severe disease and lethality are found in patients ages 60 or older, however, encephalitis is seen in individuals of all ages [1,48,62–65]. In C57BL/6 mice, POWV-LI9 entered the brain and caused neuropathology in mice of all ages. Consistent with age-dependent POWV disease seen in humans, POWV-LI9 infected mice had prolonged POWV loads in the brain and increased lethality in aged mice [48].

The cellular targets of neuroinvasive POWV have not been fully characterized, but several studies have shown POWV-positive neurons *in vivo* [44,45,66]. POWV also infects neurons and astrocyte co-cultures *in vitro* [66]. TBEV has been shown to persist in astrocytes without causing cytopathic effects, suggesting a mechanism for recurrent long-term disease seen in patients [50,67,68]. A POWV study reports the detection of persistent POWV RNA in the brains of mice [69]. POWV infection of neurons and astrocytes does not result in widespread apoptosis *in vitro* suggesting cellular reservoirs for long-term CNS disease. Further, POWV forms punctate viral structures in infected neurons *in vitro* and *in vivo* [66]. Previous FV studies have shown that the formation of similar structures in neurons can result in the loss of neuronal function and synaptic communication [70]. Disruption of such neuronal pathways may result in neurodegeneration, but this characterization requires continued studies. As does the characterization of POWV cellular targets and glial cells that contribute to the initial and delayed neuropathology seen in patients.

## Virulence factors

### Molecular determinants of POWV virulence

Viral virulence factors play a critical role in disease severity and lethality, especially factors that contribute to neurovirulence [71]. Understanding the role and molecular mechanisms of POWV virulence factors will be critical in limiting POWV disease with novel therapeutics or vaccines. Here, we discuss several POWV proteins as virulence factors including POWV E, NS1, and NS5. We also discuss host-specific factors that contribute to neurovirulence and the importance of animal models in studying these factors.

### Envelope protein (E)

FVs have three structural proteins: prM, E, and Capsid. E is the only protein exposed on the surface of mature FV virions. Therefore, E mediates viral entry into host cells by receptor-mediated endocytosis and determines cell tropism. E is also the major antigenic determinant of FVs recognized by neutralizing antibodies and immune cells, and controls immune cell activation [4,39,40,72–74]. Residues in TBEV E have been linked to the control of neurovirulence as well as alteration of host tropism range [75–80]. E is composed of three domains (I, II, III). TBEV Domain III has been associated with cell attachment and residues within Domain III appear critical for cellular entry and host range [71]. A recent POWV-LI9 study revealed that a single residue change in E Domain III (D308N) abolished viral lethality and neuroinvasion in 50-week-old mice, reflecting the avirulent phenotype [81]. This suggests that Domain III residues are critical for POWV neurotropism and lethality. Domain I also contains several sites that have been indicated in TBEV infectivity and virulence, including residues involved in E structure stability and an *N-*linked glycosylation site [76,82–84]. For attenuated vaccine development, E virulence residues of interest are those that can be targeted to prevent neurovirulence (eg. D308N in POWV LI9) but still mount an immune response for long-term immunity [71]. A better characterization of the mechanisms of POWV neuroinvasion and POWV E-specific receptors at the bite site and in the CNS will be important in vaccine development.

### Non-structural protein 1 (NS1)

Unlike structural proteins, FV non-structural (NS) proteins are not packaged into new viral particles. Instead, FV NS proteins are involved in viral replication and virion assembly. FV NS1 is translocated into the endoplasmic reticulum (ER) where it is glycosylated and dimerized [85–96]. NS1 dimers in the ER lumen form FV replication complexes with NS2A/4A/4B and are eventually secreted from infected cells as soluble hexamers [85,86,88,92,95,97–101]. Secreted DENV NS1 is detected in the blood of patients and is an indicator of viraemia [102]. Further, the secretion of NS1 systemically provides an antigen for immunogenic responses during FV infection, including during DENV, TBEV and WNV infections [103–106]. Aside from a critical role in viral replication, FV NS1 has several pathogenic functions including complement binding, severity of infection, and vascular permeability [85,86,88,89,91,95–97,107–110]. Therefore, NS1 has been identified as a FV virulence factor that may be targeted for viral attenuation [85,93,97,111,112]. Like E, NS1 contains conserved glycosylation sites that have been shown to impact FV neurovirulence by inhibiting replication [89,94,95]. The POWV NS1-N224 glycosylation site has been identified as a potential POWV attenuation residue due to its ability to influence POWV replication and cell-to-cell spread *in vitro* and neurovirulence *in vivo* [20].

### Non-structural protein 5 (NS5)

NS5 is the largest and most conserved FV protein. NS5 is the viral polymerase that contains a methyltransferase at the N-terminus and C-terminal RNA-dependent RNA polymerase [113,114]. FV NS5 proteins regulate innate immune responses through suppression of type I IFN signalling, regulation of STAT2 transcriptional responses, and downregulation of IFN receptor IFNAR1 expression [113–119]. TBEV NS5 regulation of IFN signalling was associated with viraemia, entry into the CNS, and lethality [113,116]. The enzymatic activity of NS5 and its role in viral replication and neurovirulence make NS5 a potential POWV therapeutic target. TBEV NS5 is thought to bind PDZ domains, which are modules that promote protein-protein interactions [71,119–121]. TBEV NS5 binding to PDZ is associated with IFN-I antagonism and viral replication [119–122]. This suggests disruption of NS5-PDZ interactions may promote viral clearance at the periphery and dampen neurovirulence. However, the FV NS5 PDZ motif is buried between 2 pentameric NS5 complexes that assemble into a functional decameric NS5 polymerase and may be unavailable for cell-protein interactions [123–126]. Targeting the POWV NS5-PDZ interactions with attenuated mutants may elucidate the roles of these interactions in POWV virulence and assist in the development of potential therapeutics.

### Relevance host-specific factors in studying virulence

Host-specific factors can contribute to virulence and, as a result, disease severity. FV studies have shown that neuroinvasive POWV pathogenesis is restricted by host genetic factors [59]. It is crucial to define the roles of host-specific and viral virulence factors in POWV pathogenesis. Animal models that mimic POWV disease in humans are critical in studying POWV virulence. As with any emerging virus, early POWV studies were conducted in several different animal models including *Peromyscus leucopus*, C57BL/6 mice, and BALB/c mice with various Lineage I (LB) and Lineage II strains (DTV, POWV-LI9) [20,43–45,47,48,52,127,128]. Further, studies have been conducted in mice of all different ages ranging from 4 weeks to 50 weeks and via various routes of infection [20,43–45,47,48,52,127,128]. Consistent with this, lethality in mice is reported to vary by strain, inoculation titres, and timing of POWV neuroinvasion [43,45,46,48,69,127–130]. This poses a challenge for comparing data across studies. A recent study in 10–50 week old C57BL/6 mice demonstrated POWV-LI9 infects and invades the CNS of all ages but POWV lethality increased dramatically with increased age [48]. Age is a host-specific factor that is often overlooked but plays a role in the pathogenesis of several neurovirulent FVs, including TBEV and WNV [62,63,65,131]. Other studies have reported that the addition of tick salivary gland extract to POWV inoculum enhances POWV neurovirulence and lethality. Tick saliva is thought to lessen host-immune responses at the bite site [43,128,132]. Differences in host-specific responses in the skin may control viral dissemination and neurovirulence. Together, these examples emphasize the influence of host-specific factors in POWV disease and the importance of animal models in virulence studies.

In addition, understanding POWV infection kinetics in animal models is also important in determining if there are clinical differences between Lineage I and Lineage II POWVs. Lineage I and lineage II POWVs are serologically and clinically indistinguishable in humans [15,133]. However, some studies in mice suggest that there may be differences in infection modes, neuroinvasion, and disease presentation [45]. If there are significant differences in POWV lineage pathogenesis and virulence, it could suggest varying drug targets and treatment options.

## Host immune responses to POWV

From infection at the skin surface to invasion of the neuronal compartment, neurovirulent FVs must evade a multitude of host innate and adaptive immune responses to cause encephalitic disease [134]. Here, we address the current understanding of host immune responses to POWV infection, immunopathological consequences of infection, potential POWV immune evasion strategies, and potential immune-based vaccines

### Immune responses at tick bite site

The skin is a physical barrier that acts as the first line of defence against infection [135]. Ticks insert a barbed hypostome into the skin surface, which mechanically disrupts the skin barrier, and inject saliva containing saliva-associated pathogens [47,136]. To prolong attachment and feeding, *Ixodes* saliva contains molecules that modulate wound healing and immune responses at the feeding site [14,31,137–140]. Active saliva factors promote vasodilation and inhibit pain, itching, inflammation, complement activation, and antigen processing for adaptive immune responses [141–149]. Therefore, tick saliva is not only a conduit for pathogen infection but supports viral transmission by dampening local immune responses [14,43,132,150,151]. Studies have demonstrated saliva-assisted transmission (SAT) for several tick-borne pathogens, including TBEV and POWV [43,152–155]. The specific factors that promote SAT and enhance POWV infection at the feeding site remain elusive. However, SAT factors may be potential therapeutic targets to control tick-borne FVs.

POWV can be passed to mammalian hosts in tick saliva within the first 15 minutes of tick attachment and feeding [28]. POWV tick-mouse models have demonstrated rapid cutaneous changes in gene expression and immune cell recruitment at the feeding site [47,52]. Within the first 3 hours of tick attachment, and presumably POWV-LB transmission, activated cutaneous cells induced proinflammatory chemokines IL1B, IL6, IL36A, TLR4, CCR3, and CCL2 [52]. Consistent with proinflammatory gene expression; skin histology revealed macrophage and fibroblast infiltrates present at the bite site 3–24 hours post (hp) attachment. Macrophages and fibroblasts at the bite site were positive for POWV antigen 3–24 hp attachment [44,47,156]. Separate analyses of host genetic factors have linked resistance to POWV disease with reduced viral replication in macrophages [59]. However, it is unclear if phagocytic cells at the bite site are productively infected or have engulfed and degraded POWV particles. It is worth noting that no adaptive immune cells were found at any time 3–24 hp attachment [47]. Experiments using tick salivary gland extract in POWV-LB inoculations detected POWV-LB RNA in lymph nodes of infected mice within the 24 hp infection [43]. Another study reported the presence of POWV-infected macrophages in the spleen [127,128]. Together, this suggests that immune cells may be infected by POWV at the skin interface and enable transmission to the lymph nodes and potentially the brain.

### Immune response at the neuronal compartment

The BBB microenvironment and the CNS are linked by physical proximity and direct cellular contact [157–162]. Therefore, infection and inflammation at the BBB may contribute to immune-enhanced CNS disease. POWV infection of human BBB endothelial cells and pericytes revealed induction of proinflammatory chemokines (CCL5, CXCL10, CCL20, and CXC11), antiviral interferon-B and IFN-stimulated genes (RSAD2, OASL, IFIT1/2, MX2, and ISG15) [15]. Proinflammatory chemokines CCL5 and CXCL10 are associated with T cell chemotaxis, neuroinflammation, and more severe disease during TBEV infections [163–165]. CCL5 pathway inhibition or knockout reduces neuroinflammation and promotes survival in TBEV, JEV, and WNV infections. POWV infection of pericytes also induces complement-related responses (CFB, C3, C1r and C1s) that contribute to T cell recruitment and activation. Complement factors are normally induced in the CNS in response to injury [15,166,167] and are detected in the cerebral spinal fluid of TBEV and WNV patients [168–170]. Responses elicited by POWV-infected BBB cells may promote inflammatory signalling that contributes to POWV pathogenesis at the BBB-brain interface.

Neurotropic FV disease severity has been linked to failed viral clearance and CNS inflammation [48,69]. In response to POWV infection, the CNS enters an inflammatory state characterized by encephalitis, immune cell infiltration, and widespread activation of microglia and astrocytes [2,7,11,21,27,44–48,52]. Microglia and astrocytes are CNS resident glial cells that manage neurodegenerative and neuroprotective CNS responses [171–175]. A study demonstrated that age-dependent POWV lethality is correlated with a pro-inflammatory CNS state. POWV-LI9 infects mice of all ages, enters the neuronal compartment, and activates microglial cells. However, infection is 82% lethal in aged mice (50-week-old) and 7.1% lethal in young mice (10-week-old). In response to POWV-LI9 infection, aged mice brains express a Th1 transcript profile (IFNγ, IL-2, IL-12, IL-4, TNFα, IL-6), consistent with a neurodegenerative microglial program. In contrast, young mice brains express a Th2 profile (IL-10, TGFβ, and IL-4), consistent with a neuroprotective microglial phenotype [48]. This data suggests that glial cell phenotype is critical for effective viral clearance and controlling disease severity. It is important to note that lymphocyte infiltration is also a potential mechanism of CNS damage [66]. Lymphocytic inflammation has been characterized in encephalitic POWV patients and CD8+ T cells play a role in TBEV immunopathology [14,17,57,66,176,177]. Interestingly, a comparison of Lineage I (LB) and Lineage II (DTV) POWVs noted a significant increase in CX3CR1 and AIRE genes, which suggests the recruitment of CD8+ T-cells in Lineage I POWV-infected tissues [45]. Lymphocyte recruitment to the CNS has been noted in POWV *vivo* studies [14,43–46,48]. However, further studies must be done to clarify the role of lymphocytes in POWV neuropathology.

### Immune evasion strategies employed by POWV

Flavivirus pathogenesis is dependent on the evasion of the host’s innate and adaptive immune system. FVs have been shown to use their non-structural proteins to decrease the signalling of the JAK/STAT pathway, block the complement system, and limit the recognition of pathogen-associated molecular patterns in monocytes, dendritic cells or natural killer cells [134]. Studies suggest that POWV may utilize several mechanisms to escape the host response, including IFN and neutralizing antibody evasion, macrophage infection, and entering the immune-protected neuronal compartment [15,43,47,48,81].

FVs employ many different strategies to block antiviral type I IFN signalling and dampen the innate immune response [134]. IFN Pretreatment of *in vitro* primary BBB endothelial cells and pericytes prevents POWV-LI9 infection. Yet, POWV-LI9 persistently infects hBMECs and pericytes up to 30 dpi [15]. This suggests that POWV infected hBMECs and pericytes may become resistant to IFN and ISG responses. In addition, a POWV study found altered IFN responses in an attenuated POWV strain (LI9P) compared to the virulent WT POWV-LI9 *in vitro*. Attenuated POWV-LI9P infection of hBMECs resulted in higher IFN induction and secretion 2–3 dpi, suggesting IFN may contribute to POWV clearance [81]. Downstream of IFN, the ISG tripartite motif-containing protein 5α (TRIM5α) possesses antiviral activity against several FVs. Interestingly, TRIM5α restricts the replication of several FV within the TBEV serogroup but not POWV or mosquito-borne FVs. In sensitive TBFVs, TRIM5α association with NS2B/3 directs the viral protein to proteasomal degradation. This suggests that residues in the POWV NS2B/3 protein may contribute to IFN-stimulated TRIM5α escape [178]. How POWV potentially regulates innate IFN responses has not been characterized.

Another potential mechanism POWV uses to escape immune responses is cell-to-cell spread. POWV-LI9 forms foci and appears to spread cell-to-cell in epithelial cells [15]. Focal cell-to-cell spread has been shown for several viruses, including the persistent flavivirus hepatitis C virus (HCV). Further studies are required, but cell-to-cell may allow POWV to elude neutralizing antibody responses, thus contributing to immune evasion [179–182].

Finally, studies suggest that POWV may infect immune cells like macrophages and escape immune detection as mentioned previously. This strategy works to evade the immune system two-fold. First, infection of macrophages prevents elimination by the phagocyte. Second, infection transforms the immune cell into a conduit for viral dissemination. Immune cells travel systemically to lymph nodes and can cross the BBB [59,183–185]. Therefore, infection of macrophages may provide a mechanism for POWV to invade the CNS and replicate in an immune-privileged site for prolonged periods.

### Potential immune-based therapies or vaccines

The six clinically approved TBEV vaccines are inactivated, whole-virus based vaccines that prevent the development of severe disease [186–188]. These vaccines are not effective against tick-borne POWV, and there are no clinically approved POWV immune-based therapies or vaccines^6.^ Currently, the only way to prevent POWV infection is to prevent contact with *Ixodes* vector ticks. Several vaccine strategies are being developed for POWV, including mRNA vaccines, synthetic DNA vaccines, virus-like particle (VLP) vaccines, and live-attenuated vaccines [23,189–192]. Lineage I and II POWVs share ~ 96% amino acid identity in their envelope proteins, and FV structural proteins are the antigenic determinants of neutralization [16]. Therefore, pre-membrane (prM) and Envelope domains are the targets of POWV vaccine strategies [4,39,40,72–74]. A lipid nanoparticle-encapsulated (LNP) mRNA vaccine encoding the POWV pre-M and E genes induced neutralizing antibody responses and protected mice (anti-Ifnar1-mAB treated) against lethal challenge with both Lineage I and II POWV strains. This data demonstrates the potential of an LNP-mRNA vaccine platform for the development of FV vaccines [192]. POWV VLPs self-assemble viral proteins into virus-like particles using viral pre-M and Envelope [193]. Studies have shown that POWV VLPs elicit neutralizing antibodies and CD8+ T cell responses in using murine models of disease [23,193]. Synthetic DNA vaccines are delivered by electroporation are also being explored as a vaccine option [190]. Similar to mRNA and VLPs, a single immunization of POWV prM-E DNA vaccine elicited broad B and T cell immunity and protected against lethal POWV disease in murine models [190]. mRNA, VLP, and DNA POWV vaccines require more studies and refinement but appear to be viable vaccine development options moving forward.

Live-attenuated vaccines mimic natural virus infection and, as a result, stimulate an immune response similar to natural infection [189,194]. The live-attenuated Yellow Fever Virus (YFV) vaccine (17D) provides life-long protection. How the residue changes in virulent YFV results in attenuation of YFV-17D is not fully understood. Regardless, YFV-17D demonstrates the ability to effectively vaccinate an arthropod-borne FV with a live-attenuated vaccine. Several studies have designed clinically approved FV vaccines using YFV-17D backbone the prM-E domain of the desired FV (eg. DENV, JEV, WNV) [195,196]. A live-attenuated POWV-DTV vaccine using a modified YFV 17D vaccine backbone with POWV prM-E substitutions has been similarly designed. In a murine model, immunized POWV-infected mice were significantly protected from the POWV diseases with a 70% survival rate [189,194]. Although effective, this live-attenuated POWV vaccine requires further attenuation to increase efficacy and safety before moving to a clinical setting. Overall, live-attenuated POWV vaccine programs warrant further studies. Further, YFV-17D was generated through ~ 240 passages of WT YFV in mouse and chicken tissues and cell lines [72,197–199]. Therefore, POWV itself has the potential to be live attenuated through persistent live passaging like the YFV-17D. Similarly, a recent study developed a live attenuated POWV strain (POWV-LI9P) by serially passaging POWV-LI9 VeroE6 cells. Attenuated POWV-LI9P fails to cause neurovirulence lethality *in vivo* and protects mice from lethal POWV-LI9 challenge, suggesting the potential for a live attenuated POWV vaccine platform [20]. Despite challenges in vaccination, POWV neuroinvasion and neurovirulent FV immune-enhanced disease emphasize the need for an effective and safe vaccine to control POWV disease.

## Future directions

In Northern Eurasia, endemic TBEV is the causative agent of 5000–10,000 cases of encephalitis each year, with fatality rates ranging from 1–20% determined by the specific TBEV subtype. Similarly, encephalitic POWV cases have a > 10% fatality rate, and 50% of survivors suffer from persistent neurologic symptoms [1]. Currently, there are no clinically approved POWV immune-based therapies or vaccines, and the only method of prevention of POWV infection is to avoid contact with tick vectors [189]. The increased prevalence of POWV disease in the NE United States and the lack of effective POWV treatments only highlight the importance of continued research for this emergent infectious disease [1]. Here, we address priority areas of POWV investigation as it pertains to therapeutic and vaccine development.

### POWV pathogenesis from bite site to brain

The development of POWV therapeutics is dependent on a detailed analysis of POWV replication, spread, and neuroinvasion *in vivo*. At the bite site, specific POWV cellular targets and immune responses remain to be characterized and are likely critical in understanding POWV spread to the brain. POWV-LB studies detect POWV RNA in lymph nodes 1 dpi, suggesting immune responders are productively infected at the tick bite site and disseminate infection to lymph nodes at early time points. Several immune cells at the skin interface could contribute to detection in the lymph nodes (macrophages, monocytes, dendritic cells) and POWV-LB antigen has been detected in macrophages and fibroblasts [43]. Although, it is unclear if these phagocytic cells are productively infected or have engulfed POWV particles. As early as 5 dpi, POWV is detected in the brain, however, POWV neuroinvasive and neurovirulence mechanisms remain unknown [14,43,48,128]. Further, the CNS cellular targets of POWV remain to be determined but likely contribute to POWV lethality. A holistic understanding of the cellular players from bite site to brain may help elucidate prophylactic targets and approaches for preventing and treating neuroinvasive POWV infection.

### Age-dependent neuropathology targets

POWV infection results in more severe disease in individuals >50 years old [7]. Aged POWV-infected mice maintain prolonged CNS viral loads and higher lethality rates (80%) compared to their young counterparts (<10% lethality) [48]. Several determinants may influence age-dependent POWV lethality. Glial cell activation is associated with neuronal cell death and TBEV, WNV, and JEV encephalitis [68,200,201]. Neuroinvasive POWV activates glial cells in murine brains of all ages. However, glial cell activation alone is insufficient to promote POWV clearance from the brains of aged mice [48]. Viral clearance may be controlled by age-dependent differences in microglial inflammatory profiles during neuroinvasive POWV infection or glial cell senescence. Th2 cytokine profiles in the brain are associated with a neuroprotective microglial phenotype that promotes cell repair. In contrast, Th1 inflammatory profiles promote a neuroinflammatory microglia phenotype related to neurodegenerative diseases like Alzheimer’s [171,174,202,203]. POWV induces neuroprotective Th2 cytokine responses in young mice brains and neurodegenerative Th1 responses in aged mice brains. Additionally, senescence likely negatively contributes to a neurodegenerative phenotype in aged mice [48]. Aging decreases regenerative neuronal stem cells and increases senescent microglia in the brain, resulting in a pro-inflammatory state [171,174,202,203]. However, how senescence, pro-inflammatory cytokines or glial cell responses contribute to age-dependent POWV lethality remains to be examined. Further studies may inform drug targets and treatment options for resolving age-dependent POWV disease in the elderly.

### Reverse genetics approaches

Reverse genetics systems can be used to genetically modify POWV and create infectious POWV recombinant mutants of interest. FV single-strand RNA encodes one large polyprotein that is co-translationally cleaved into several nonstructural and three structural proteins (Capsid, Envelope, NS1) [4]. Mutations in FV structural proteins can impact FV virulence and, therefore, are of interest for POWV research. Circular polymerase extension reactions (CPER) have been used to investigate virulence determinants for several FV (WNV, ZIKV, YFV, JEV, DENV), including POWV NS1 [20,204–209]. CPER-generated recombinant NS1 POWVs were attenuated, with reduced replication kinetics, reduced NS1 secretion, and 80% survival in mice [20]. Modification of virulence factors like NS1 or E can help elucidate the roles of these proteins in POWV pathogenesis. Further, the attenuation of infectious CPER mutants rationalizes using CPER generation of live attenuated POWVs for vaccine development. The efficacy and lifelong immunity of the YFV live attenuated vaccine supports the development of a POWV live attenuated vaccine. Moving forward, CPER approaches can be used to quickly generate robust libraries of recombinant POWVs to study virulence factors and live attenuated recombinant POWV vaccine strains.

## Conclusions

Tick-borne FVs are associated with severe encephalitic disease with long-term neurologic implications for patients. The continued threat of endemic tick-borne FVs emphasizes the importance of prophylactics and patient treatment plans. As emergent POWV case numbers continue to rise and *Ixodes* tick vector distribution expands, so does the threat of endemic tick-borne encephalitis in North America. In recent years, POWV awareness and research have improved our general knowledge of this tick-borne pathogen. However, a detailed understanding of POWV pathogenesis, including cellular targets, virulence factors, and immune response, from the bite site to neuroinvasion of the brain remains to be defined. Increased disease severity in aged individuals underscores the importance of age-dependent POWV disease models when studying POWV pathogenesis and potentially other FVs. An improved understanding of POWV virulence in conjunction with current POWV reverse genetics systems creates the potential to generate robust recombinant POWV libraries and live attenuated POWV vaccines. As the community gains more insight into neurovirulent POWV it will permit the development of POWV vaccines and therapeutics and may provide a broader understanding of tick-borne FVs.

## Accessibility of data

Data sharing does not apply to this article. No new data has been created.
